# An Efficient Elastic Net with Regression Coefficients Method for Variable Selection of Spectrum Data

**DOI:** 10.1371/journal.pone.0171122

**Published:** 2017-02-02

**Authors:** Wenya Liu, Qi Li

**Affiliations:** School of Control Science and Engineering, Dalian University of Technology, Dalian, China; Jilin University, CHINA

## Abstract

Using the spectrum data for quality prediction always suffers from noise and colinearity, so variable selection method plays an important role to deal with spectrum data. An efficient elastic net with regression coefficients method (Enet-BETA) is proposed to select the significant variables of the spectrum data in this paper. The proposed Enet-BETA method can not only select important variables to make the quality easy to interpret, but also can improve the stability and feasibility of the built model. Enet-BETA method is not prone to overfitting because of the reduction of redundant variables realized by elastic net method. Hypothesis testing is used to further simplify the model and provide a better insight into the nature of process. The experimental results prove that the proposed Enet-BETA method outperforms the other methods in terms of prediction performance and model interpretation.

## Introduction

Spectrum data is always used for quality prediction of important product or prediction of solution concentrations which are hard to measure in real industry process, especially in chemical processes. Near-infrared (NIR) spectroscopy, as a non-destructive, rapid and reliable analytical technique, has been widely used in many industry processes. However, NIR spectrum data always suffers from background variation, noise and colinearity[1]. A mass of data with hundreds of predictors is collected with many redundant variables contained, and those redundant variables contain more noise than quality-related information. Adding too many redundant variables into the regression model can lower the prediction accuracy, so variable selection plays an important role to deal with spectrum data. By identifying the key variables, variable selection can improve the prediction performance of the built model, reduce the model complexity and computation load, and provide a better insight into the nature of the process.

Stepwise regression (SR), partial least squares (PLS), least absolute shrinkage and selection operator (Lasso) and elastic net (Enet) are representative feature selection methods, and their regression coefficients carry nonnegligible information. Principle component analysis (PCA) and PLS are mostly used in dimension reduction for their simplicity and effectiveness[2], and Subsequently many variable selection methods based on PLS are proposed, such as PLS based on variable importance in projection (PLS-VIP)[3,4], PLS with regression coefficients (PLS-BETA)[5], genetic algorithm combined with PLS (GA-PLS)[6], uninformative variable elimination combined with PLS (UVE-PLS)[7], and so on. PLS-VIP is well-known for its simple implementation and cheap computation, but it is affected by variable correlation and sensitive to tuning parameter; PLS-BETA is insensitive to training data and only has one parameter to tune, but it is a little sensitive to tuning parameter; GA-PLS could escape from local optima due to randomized search, but it requires expensive computation; UVE-PLS is insensitive to tuning parameter, but it is strongly affected by the magnitude of variable correlation. Stepwise regression (SR) is popular for its easy interpretation between the results and tuning parameters, especially for forward stepwise selection (FSS), but SR may be trapped in local optima[8].

Feature selection methods can be divided into two categories: variable selection methods and variable projection methods. Variable selection methods like SR and Enet, aim to select part of the original variables to build a model, and variable projection methods like PCA and PLS, aim to project the original variables on some specific directions and obtain a group of new variables. Spectrum data, however, contains hundreds or even thousands of spectrum variables, and uninformative variables dominant an immeasurable proportion. Redundant variables always lead to overfitting, a low prediction accuracy and the increase of calculation load. The key of projection methods is finding a reliable projection direction, however, the calculation of projection direction is sensitive to training data. So too many redundant variables will badly affect the selection of projection direction, and projection methods may not perform well in dealing with spectrum data. Variable selection methods, like shrinkage methods show a great advantage facing with spectrum data.

Shrinkage methods[9] are based on original least squares (OLS), like ridge regression, lasso[10–11] and Enet[12]. LARS[13] and LARS-EN[12] are used to solve the entire lasso and Enet solution path respectively. And multiway elastic net (MEN) is used to deal with three-dimensional data for batch process[14]. Enet can shrink the coefficients of redundant variables exactly to zero, however, PLS adds all the process variables into the model regardless of the uninformative variables. So the coefficients of Enet are more stable and reliable compared with those of PLS, and we can use them to further select quality-related variables and reduce model complexity. In this study, an elastic net with regression coefficients (Enet-BETA) method is proposed to perform variable selection based on the regression coefficients of Enet. Two case studies are given to demonstrate its feasibility by comparing with PLS, PLS-BETA, FSS and Enet methods and the prediction performance is also improved apparently.

The remainder of this paper is organized as follows: Section 2 briefly reviews different variable selection methods and Section 3 introduces the proposed Enet-BETA method. Section 4 compares the performance of different variable selection methods by two industrial case studies. And the conclusions will be drawn in Section 5.

## Preliminary

In this section, four variable selection methods are briefly introduced as follows.

### Partial least squares

PLS is a well-known multivariate statistical technique for modeling the relationship between *p* process variables, *X*_(*n*×*p*)_, and *l* product quality variables, *Y*_(*n*×*l*)_, with *n* samples, as shown in (1),

$$\begin{array}{l} X=TP^{T}+E\\ Y=TQ^{T}+F\\ T=XW\;{\left(P^{T}W\right)}^{-1} \end{array}$$ (1)

where *T*_(*n*×*h*)_, *P*_(*p*×*h*)_ and *E*_(*n*×*p*)_ are the score, loading, and residual matrices of *X*; *Q*_(*l*×*h*)_ and *F*_(*n*×*l*)_ are the loading and residual matrices of *Y*; *W*_(*p*×*h*)_ is the weight matrix and *h* is the number of principle components which can be obtained by K-fold cross validation. With a PLS model, the prediction of a new sample can be obtained as follows:

$$\begin{array}{l} \hat{Y}=X_{new}\beta_{PLS}\\ \beta_{PLS}=W{\left(P^{T}W\right)}^{-1}Q^{T} \end{array}$$ (2)

where $\hat{Y}$ is the prediction of the new sample, and *β*_*PLS*_ is the regression coefficient vector of the built PLS model.

The goal of PLS is to maximize the covariance between the principle components of *X* and *Y*. When the original variables are highly correlated, redundant, noisy, and of high dimensionality, PLS can obtain a group of orthogonal scores by project *X* and *Y* on some orthogonal directions respectively, and the scores would contain sufficient process information of *X* and predictive information of *Y*. PLS model is more stable than the model built upon the original variables, since the regression is done on the scores instead of the original variables.

### PLS with regression coefficients

PLS with regression coefficients named PLS-BETA[5] directly utilizes the regression coefficients estimated by PLS. The significant variables are selected according to the magnitude of the absolute values of regression coefficients. The estimation $\hat{y}$ is expressed as follows:

$$\hat{y}=T{\left(T^{T}T\right)}^{-1}Ty=X\beta_{PLS}$$ (3)

where the regression coefficients vector is described as

$$\beta_{PLS}=W{\left(P^{T}W\right)}^{-1}{\left(T^{T}T\right)}^{-1}y$$ (4)

$$\frac{\left\|\beta_{select}\right\|}{\left\|\beta_{PLS}\right\|}>\alpha$$ (5)

The input variables can be selected individually in descending order of the magnitude of *β*_*PLS*_, until Eq (5) is achieved, where *β*_*select*_ denotes the vector of the regression coefficients corresponding to the selected variables and 0 < *α* ≤ 1.

### Stepwise regression

SR is a standard procedure for variable selection which is based on the procedure of sequentially adding the predictors into the model one at a time. Forward stepwise selection (FSS)[15] starts with the intercept, and then sequentially adds the predictor that most improves the fit into the model. FSS produces a sequence of models indexed by *k*, the subset size, which must be determined. Backward stepwise selection (BSS) starts with the full model, and sequentially deletes the predictor that has the least impact on the fit. An advantage of FSS for a large number of highly correlated variables is that the *X*^*T*^ *X* matrix does not need to be inverted, while BSS can only be used when *n* > *p* (the number of samples is larger than that of variables).

In the above two methods, the number of predictors retained in the final model is determined by Bayesian information criterion (BIC), which will be presented in the next section.

### Least absolute shrinkage and selection operator

We consider the usual linear regression model: given *p* predictors *x*_1_,…,*x*_*p*_, the response *y* is predicted by

$$\hat{y}={\hat{\beta }}_{0}+x_{1}{\hat{\beta }}_{1}+\ldots +x_{p}{\hat{\beta }}_{p}$$ (6)

A model fitting procedure produces the vector of coefficients $\hat{\beta }=\left({\hat{\beta }}_{0},\ldots ,{\hat{\beta }}_{p}\right)$. Ordinary least squares (OLS) estimation is obtained by minimizing the residual sum of squares, but OLS often does poorly in both prediction and interpretation. Penalization techniques have been proposed to improve the performance of OLS[16]. For example, ridge regression minimizes the residual sum of squares subject to a bound on the *L*_2_-norm of the coefficients. However, ridge regression cannot produce a parsimonious model, because it always keeps all the predictors in the model.

Lasso is a penalized least squares method which imposes an *L*_1_-norm penalty on the regression coefficients, and it is shown as follows:

$${\hat{\beta }}_{lasso}=\underset{\beta }{\arg\;\min}\;{\left\|y-\sum_{j=1}^{p}x_{j}\beta_{j}\right\|}^{2}$$ (7)

subject to

$${\left\|\beta \right\|}_{1}\leq t$$ (8)

Owing to the nature of *L*_1_-norm penalty, the lasso does both continuous shrinkage and automatic variable selection simultaneously[17]. As variable selection becomes increasingly important in modern data analysis, lasso is much more appealing owing to its sparse representation. However, lasso also has some limitations.

1. In the *p* > *n* case, lasso selects at most *n* variables before it saturates owing to the nature of the convex optimization problem.
2. If there are a group of highly correlated variables, lasso will select only one variable but does not care which one is selected. So lasso fails to do group selection.

## Proposed Variable Selection Method

### Enet method

Similar to lasso, Enet simultaneously does automatic variable selection and continuous shrinkage, and it can select groups of correlated variables[18]. Enet shrinks the regression coefficients by combining *L*_1_-norm penalty (lasso) and *L*_2_-norm penalty (ridge) together.

$${\hat{\beta }}_{enet}=(1+\frac{\lambda_{2}}{n})\left\{\underset{\beta }{\arg\;\min}\;{\left\|y-\sum_{j=1}^{p}x_{j}\beta_{j}\right\|}^{2}+\lambda_{1}{\left\|\beta \right\|}_{1}+\lambda_{2}{\left\|\beta \right\|}_{2}^{2}\right\}$$ (9)

The *L*_1_-norm part of the penalty generates a sparse model by shrinking some regression coefficients exactly to zero. The *L*_2_-norm part of the penalty removes the limitation on the number of selected variables, encourages grouping effect, and stabilizes the *L*_1_ regularization path[19]. An efficient algorithm LARS-EN [15] is proposed to compute the entire Enet regularization paths with the computational effort of a single OLS fit.

### Enet-BETA method

Similar to PLS-BETA, Enet-BETA directly utilize the regression coefficients estimated by Enet, and the significant variables are selected according to the magnitude of the absolute values of regression coefficients of Enet.

As we all know, spectrum data with hundreds of variables contains lots of redundant variables which can reduce the prediction accuracy. So shrinkage methods are indispensable to deal with spectrum data. Although PLS is a powerful method, but the coefficients are nonzero for every variable. That is to say, all the available variables are used to build the PLS model. In this way, the model is more or less effected by the redundant variables, and it is also easy to prone to overfitting. Enet method can keep high correlated variables simultaneously into or out of the built model, but the process variables of spectrum data always suffer high correlation between them, so Enet can’t always get a sparse enough regression model when dealing with spectrum data. Different with PLS, Enet can efficiently shrink the regression coefficient of redundant variables exactly to zero. So Enet model is more stable than PLS model, and there is no doubt that the regression coefficients of Enet model are more reliable than those of PLS model. Enet-BETA method is not easy to prone to overfitting due to the reduction of redundant variables. On the theoretical basis mentioned above, we can conclude that Enet-BETA is more efficient than PLS-BETA. Enet-BETA method can reduce the model complexity and computation load, lower the measurement cost, and provide a better insight into the nature of the process.

Elastic net method is able to select groups of highly correlated variables, but the correlation between most variables all reach up to 0.95 which leads to the inefficiency of elastic net to get a sparse model and makes the results hard to interpret. The proposed Enet-BETA method can obtain a sparser model based on the regression coefficients of elastic net, and a small part of variables is remained to build an accuracy model which makes it explicit to find that the quality is affected by which process variables, so the interpretability will be improved by the proposed Enet-BETA method.

The number of predictors retained in the final model is determined by root-mean-square error of prediction (*RMSEP*), a criteria used to evaluate prediction accuracy, as shown in (10).

$$RMSEP=\sqrt{\frac{{\sum_{i=1}^{n}\left(y_{i}-{\hat{y}}_{i}\right)}^{2}}{n}}$$ (10)

Where $\hat{y}$ is the predicted value of response *y*, and *n* is the number of samples.

In order to obtain a sparser and more explanatory model, we use hypothesis testing (HT) to reduce the number of selected variables by sacrificing the prediction accuracy.

$$\frac{RMSEP(i^{*})}{RMSEP(i)}\geq \lambda$$ (11)

Where i^*^ is the best number of selected variables. *λ* is confidence level, and we set *λ* = 0.9 in the two case studies. We have *i* < i^*^, and *i* is the number of selected variables. For clarity, the procedure of the proposed Enet-BETA method is summarized as below. Enet-BETA method is described as following steps.

Step 1: Normalize the original datasets *X*_(*n*×*p*)_ and *Y*_(*n*×1)_ to zero mean and unit variance.
Step 2: Perform Enet on normalized datasets and adjust two parameters to get the regression coefficients *β*.
Step 3: Sort the absolute value of regression coefficients *β* in a descending order and mark it as β^*^, then sequentially add the predictor which has the largest magnitude of β^*^ to form a new training dataset and perform Enet on the selected variables. So we can get *p* models.
Step 4: Calculate the *RMSEP* index of *p* models.
Step 5: Select the minimum *RMSEP* and mark the relevant subset size as i^*^.
Step 6: According to hypothesis testing, select a sparser model by sacrificing prediction accuracy.

## Case Studies

### Criteria

In order to evaluate the performance of different variable selection methods, several performance indices have been proposed in the literature. Akaike's information criteria (AIC) and Bayesian information criterion (BIC) are two common information criteria based on maximum likelihood function. Normalized mean square error (NMSE) and coefficient of determination (*R*^2^) are data-driven criteria based on the predicted qualities. And k-fold cross validation is mostly used to determine the best number of principle components in PLS.

#### AIC and BIC

AIC has the advantage of testing the significance of different model specifications. Sakamoto proposed an alternative to AIC, called BIC, which is also a tool of selecting the best model. A lower AIC or BIC value indicts a better model. They are defined as follows.

AIC=−2ln(L)+2k (12)

BIC=−2ln(L)+kln(n) (13)

Where *L* is the maximized value of the likelihood function, *k* is the number of selected variables, and *n* is the sample size. BIC enforce stronger penalty on the number of selected variables than AIC, so in this paper, we use BIC to select the best number of selected variables in FSS and the best regression coefficient vector in Enet.

#### NMSE

NMSE is a similar criterion to RMSEP, which also measures the prediction accuracy of the built model. NMSE can be calculated as follows:

$$NMSE=\frac{{\sum_{i=1}^{n}\left(y_{i}-{\hat{y}}_{i}\right)}^{2}}{y_{i}{}^{2}}$$ (14)

Where $\hat{y}$ is the predicted value of response *y*, and *n* is the number of samples.

#### Coefficient of determination

*R*^2^ measures how well the data fits the model, and it can be calculated as follows.

$$R^{2}=1-\frac{SSR}{SST}$$ (15)

Where $SSR=\sum_{i=1}^{n}{\left(y_{i}-{\hat{y}}_{i}\right)}^{2}$ is the sum of squared residual, $SST=\sum_{i=1}^{n}{\left(y_{i}-\bar{y}\right)}^{2}$ is the total sum of squares, and $\bar{y}$ is the average of *y*. The range of *R*^2^ is between 0 and 1. If *R*^2^ is closer to 1, it means that the model is more accurate.

In order to evaluate the performance of the proposed Enet-BETA method, two case studies are used to demonstrate its effectiveness in this paper. The experiment of different variable selection methods is calculated with Matlab R2015b, and the computer configuration is described as follows, CPU: 3.2GHz, RAM: 8.00GB, Windows 7. Two datasets all carry the character of multicollinearity, and the correlation coefficients between most variables all reach up to 0.95 which greatly increases the difficulties of variable selection.

### Case one: Multivariate calibration of wheat kernel data

This dataset is wheat kernel, which relates to the percentage of protein concentration. This NIR spectrum data is recorded at 100 wavelengths across the region 850–1050 nm. This dataset has been divided into a training set of 415 samples and a test set of 108 samples, and it is widely used as a benchmark dataset. This dataset is publicly available on http://www.models.life.ku.dk/wheat_kernels.

In this experiment, we compare Enet-BETA method with PLS, PLS-BETA, Enet and FSS methods. The comparison of the five different variable selection methods is tabulated in Table 1. In Table 1, the NOVS is the number of selected variables. *RMSEP*_*te*_, *NMSE*_*te*_ and *R*^2^ indices are used to evaluate the accuracy of different models, and the coefficients of determination of training data and testing data are expressed as *R*^2^_*tr*_ and *R*^2^_*te*_ respectively. From Table 1, we can see that the proposed Enet-BETA method outperforms the other four methods apparently. In the PLS model, the number of principle components is determined to be 9 according to 9-fold cross validation. Based on the coefficients of developed PLS model, PLS-BETA method is performed to select important variables and 9 variables are selected. Although the prediction accuracy is improved compared with PLS, it is relatively lower than Enet and proposed Enet-BETA method. FSS selects 19 variables from the original 100 variables, but the *RMSEP*_*te*_ and *NMSE*_*te*_ index is the highest compared with others. Enet performs well by shrinking the regression coefficients of partial redundant variables exactly to zero and selects only 40 important variables to build the regression model. Based on the 40 variables selected, Enet-BETA method further selects 14 variables to build a new model, and gets the highest prediction accuracy. We can see apparently that Enet-BETA can get the simplest model and an accurate enough prediction effect.

**Table 1:** Comparison of the results using six different variable selection methods.

Methods	NOVS	*RMSEP*_*te*_	*NMSE*_*te*_	*R*^2^_*tr*_	*R*^2^_*te*_	*Time*
PLS	100	0.5910	0.4538	0.8641	0.8930	16.878
PLS-BETA	9	0.5838	0.5193	0.8664	0.8875	18.003
FSS	19	0.5912	0.4564	0.8643	0.8847	50.545
Enet	40	0.5275	0.3502	0.8900	0.9082	21.171
Enet-BETA	14	0.5150	0.3156	0.8721	0.9125	29.541

NOVS, number of selected variables.

*RMSEP*_*te*_, root-mean-square error of prediction for the test data.

*NMSE*_*te*_, normalized meansquare error of prediction for the test data.

*R*^2^_*tr*_, the coefficients of determination of training data.

*R*^2^_*te*_, the coefficients of determination of test data.

*Time(s)*, the running time of the variable selection method

From Table 1, we can see that the proposed Enet-BETA method is more time-consuming compared with PLS, PLS-BETA and Enet, but the modeling part is just performed offline. It is acceptable to get a more accuracy offline model regardless of the time-consuming fact. The online application won’t be time-consuming at all because of the remove of redundant variables.

**Fig 1:**
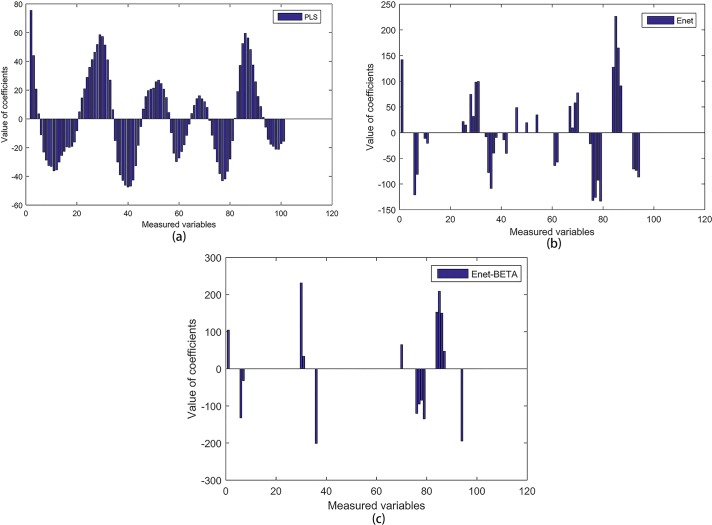
Plot of coefficients obtained by PLS, Enet and Enet-BETA. **(a)** This figure shows the regression coefficients of 100 variables obtained by PLS, and it means that PLS selects all the process variables into the regression model. **(b)** This figure shows the regression coefficients of 100 variables obtained by Enet, and we can see that part of coefficients are shrank to be zero, which means that Enet selects part of the process variables into regression model. **(c)** This figure shows the regression coefficients of 100 variables obtained by Enet-BETA, and it’s clear that only a small part of variables are selected into regression model.

**Fig 2:**
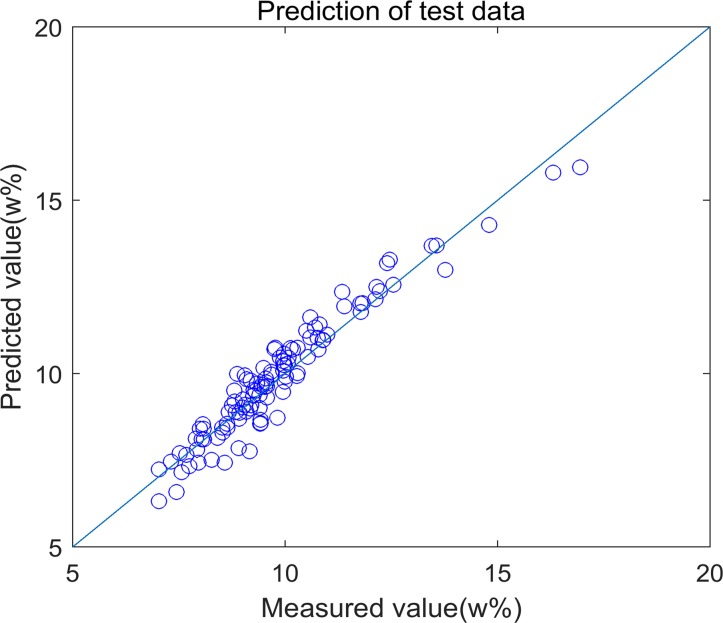
Plot of predicted vs. measured value of Enet-BETA. The scatter plot depicts the prediction accuracy of the built model. The x axis represents the measured value of the percentage of protein concentration and the y axis represents the predicted value by the regression model.

The regression coefficients of PLS, Enet and Enet-BETA are showed in Fig 1. We can see that the coefficients of Enet are sparser than those of PLS after shrinking the redundant ones to zero. Enet-BETA can get the simplest model and provide a better insight into the nature of process. The predicted concentration vs. the measured concentration is plotted in Fig 2 from which we can see that the model built by Enet-BETA method can predict the qualities in a high accuracy.

### Case two: Multivariate calibration of crystallization spectrum data

**Fig 3:**
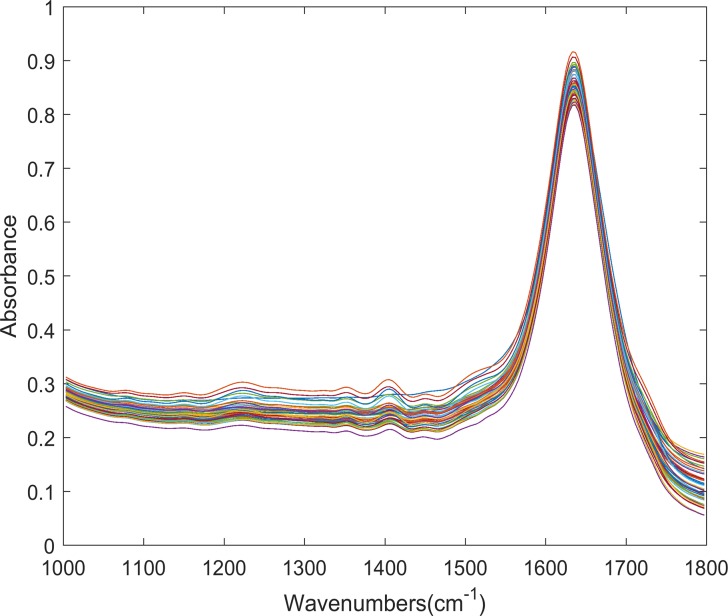
Plot of predicted vs. measured value of Enet-BETA. This plot depicts the spectra of different LGA solution concentrations at 9.0, 15.0, 21.0, 27.0, 33.0, 39.0 g/L, and it’s clear that there exist high-colinearity between process variables.

The second spectrum dataset is the crystallization, which relates to the concentration of LGA. It is measured by ATR-FTIR spectroscopy at different LGA solution concentrations and temperatures. The details of this experimental set-up are also introduced in Qi’s paper[20]. The spectrum data is recorded at 215 wavelengths across the region 1000–1800. This dataset is divided into a training set of 227 samples and a test set of 75 samples. The solution concentration is measured at 9.0, 15.0, 21.0, 27.0, 33.0, 39.0 g/L and the temperature ranges from 15 to 75°C. The ATR-FTIR spectra of different LGA solution concentrations is plotted in Fig 3, from which we can see that collinearity exists seriously. This increases the difficulty of building the regression model.

The comparison of the six different variable selection methods is tabulated in Table 2. In Table 2, the NOVS is the number of selected variables. *RMSEP*_*te*_, *NMSE*_*te*_ and *R*^2^ indices are used to evaluate the accuracy of different models, and the coefficients of determination of training data and test data are expressed as *R*^2^_*tr*_ and *R*^2^_*te*_.

**Table 2:** Comparison of the results using six different variable selection methods.

Methods	NOVS	*RMSEP*_*te*_	*NMSE*_*te*_	$R_{tr}^{2}$	$R_{te}^{2}$	*Time*
PLS	216	0.2221	0.0156	0.9995	0.9995	17.476
PLS-BETA	49	0.1450	0.0084	0.9946	0.9955	20.346
FSS	25	0.1614	0.0121	0.9998	0.9997	70.564
Enet	147	0.1396	0.0077	0.9999	0.9998	22.302
Enet-BETA	32	0.1283	0.0069	0.9998	0.9997	30.461

NOVS, number of selected variables.

*RMSEP*_*te*_, root-mean-square error of prediction for the test data.

*NMSE*_*te*_, normalized meansquare error of prediction for the test data.

*R*^2^_*tr*_, the coefficients of determination of training data.

*R*^2^_*te*_, the coefficients of determination of the first test data.

*Time(s)*, the running time of the variable selection method.

From Table 2, we can see that the proposed Enet-BETA method outperforms other methods apparently. The number of principle components of PLS model is determined to be 7 via 9-fold cross validation. Each variable has a regression coefficient with a certain nonzero value, and it results in overfitting for too many redundant variables are involved into the model, especially for the second test dataset. From Table 2, we can see apparently that the PLS-BETA model built based on PLS coefficients performs even worse in predicting the qualities of the second test data. Although FSS method obtains a sparse enough model, not only the prediction ability is very poor, but overfitting also exists. Enet gets a relatively sparse model by selecting 147 significant variables, but apparently it also contains redundant variables. The proposed Enet-BETA method further selects significant variables based on the regression coefficients of Enet, and 32 variables are selected which can reach the highest prediction accuracy. We can see apparently that Enet-BETA can get the simplest model and an accurate enough prediction effect. From a comprehensive comparison, we can get that the model built by Enet-BETA method can predict the quality in a high accuracy with a sparse enough model. The advantage of Enet-BETA method relates to the sparsity of Enet method after shrinking some redundant coefficients to zero.

**Fig 4:**
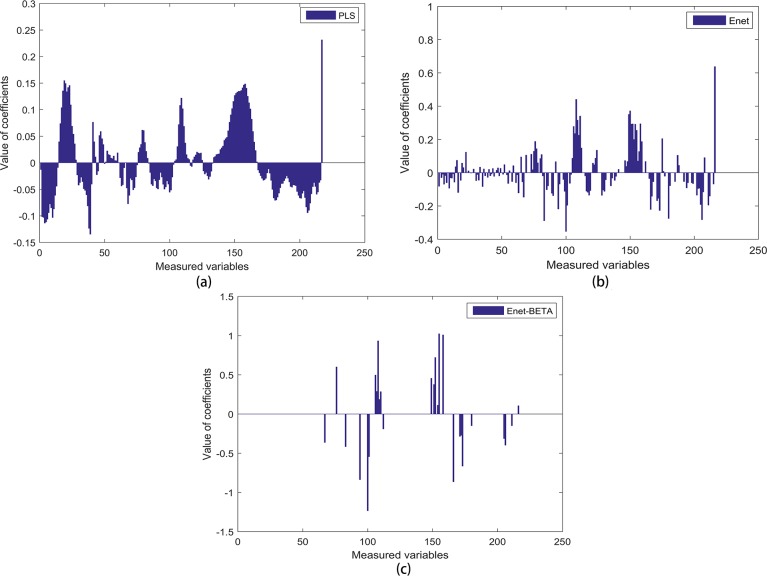
Plot of coefficients obtained by PLS, Enet and Enet-BETA. **(a)** This figure shows the regression coefficients of 216 variables obtained by PLS, and it means that PLS selects all the process variables into the regression model. **(b)** This figure shows the regression coefficients of 216 variables obtained by Enet, and we can see that part of coefficients are shrank to be zero, which means that Enet selects part of the process variables into regression model. **(c)** This figure shows the regression coefficients of 216 variables obtained by Enet-BETA, and it’s clear that only a small part of variables are selected into regression model.

**Fig 5:**
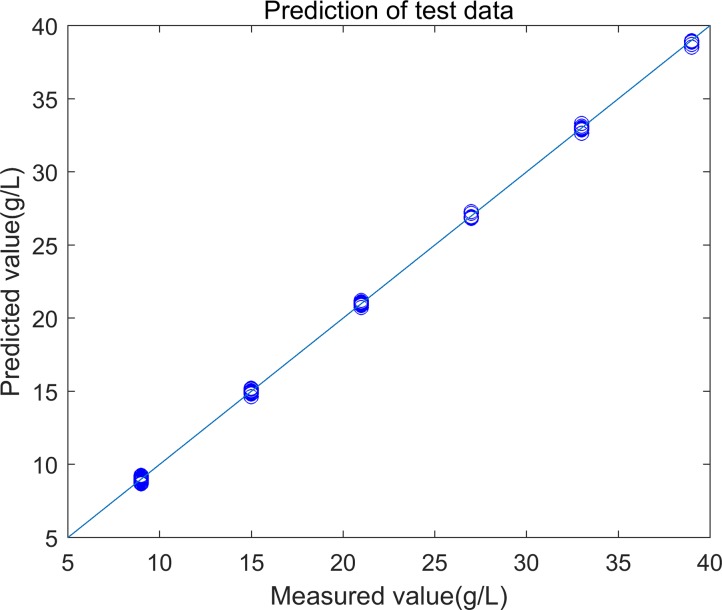
Plot of predicted vs. measured value of test data. The scatter plot depicts the prediction accuracy for the first test data with. The x axis represents the measured value of the percentage of protein concentration and the y axis represents the predicted value by the regression model.

The coefficients of PLS, Enet and Enet-BETA are plotted in Fig 4. It shows that Enet model is sparer than PLS model, but Enet-BETA can get the sparsest model. Enet-BETA can provide a better insight into the nature of process and find out the real quality-related variables. The predicted concentration vs. the measured concentration is plotted in Fig 5 which shows the prediction results of the test data.

## Conclusion

In this paper, an Enet-BETA method has been proposed to build a stable and accuracy regression model via variable selection. This method can not only select important variables to make the response easy to interpret, but also can improve the stability and feasibility of the built model. Then two case studies are given to demonstrate the effectiveness of proposed method by comparing with the other four variable selection methods. Meanwhile, Enet-BETA method reflects the advantage of shrinkage methods.
